# Comparing the antibacterial efficacy and functionality of different commercial alcohol-based sanitizers

**DOI:** 10.1371/journal.pone.0282005

**Published:** 2023-03-27

**Authors:** Kaiyang Lim, Wei Yi Li, Afrah Dinata, En Ting Ho

**Affiliations:** 1 ES-TA Technology Pte Ltd, Singapore, Singapore; 2 Singapore Institute of Technology, Food, Chemical and Biotechnology Cluster, Singapore, Singapore; University of Jeddah, SAUDI ARABIA

## Abstract

The use of alcohol-based sanitizers has been recommended as an effective alternative to clean hands, especially in the case when hand washing is not doable. This is especially critical with the COVID-19 pandemic, where personal hygiene is an important factor to deter the spread of the virus. This study assesses and evaluates the differences in antibacterial efficacy and functionalities of five different commercial alcohol-based sanitizers with different formulations. All sanitizers were able to provide instant sanitization functionality, effectively killing 5x10^5^ CFU/mL of inoculated bacteria. However, comparing pure alcohol-based sanitizers against alcohol-based sanitizers with a secondary active ingredient demonstrated that the addition of a secondary active ingredient enhanced the effectiveness and functionalities of the sanitizers. Alcohol-based sanitizers with secondary active ingredients demonstrated a more rapid antimicrobial mode of action, eradicating all 10^6^ CFU/mL of bacteria within 15 seconds of contact, in contrast to the 30 min for purely alcohol-based sanitizers. The secondary active ingredient also provided additional anti-biofilm functionality to prevent opportunistic microbes from attaching and proliferating on the treated surface, leading to serious biofilm formation. On top of that, treatment of surfaces with alcohol-based sanitizers with secondary active ingredients also imparted prolonged antimicrobial protection to the surface lasting up to 24 h. On the other hand, purely alcohol-based sanitizers do not seem to possess such quality with the treated surface being vulnerable to microbial contamination within minutes after application. These results highlighted the benefits of adding a secondary active ingredient in sanitizer formulation. However, care needs to be taken to evaluate the type and concentration of antimicrobial agents chosen as the secondary active ingredient.

## Introduction

Since the explosion of the COVID pandemic, the use of sanitizers has become the new normal [1]. According to a recent survey by For Markets, the global hand sanitizer market is projected to grow from USD 1.2 billion in 2019 to USD 2.14 billion by 2027, at a compound annual growth rate of 7.5% during the forecast period 2019–2027. The demand for hand sanitizers has faced drastic growth of 16 times comparing between December 2019 and March 2020. Commercially, there are two types of sanitizers being sold in the market, namely alcohol-based and water-based sanitizers. Of which, only alcohol-based sanitizers are officially recommended by the United States Centers for Disease Control and Prevention (CDC) as well as the World Health Organization (WHO) as an alternative to conventional soap and water for cleaning hands [2,3]. Based on CDC’s requirement, an effective alcohol-based sanitizer should contain at least 60% alcohol [3–5]. A study by *Masaru et al*. showed that the effect of ethanol concentration on bactericidal potency follows a U-shape profile, where antibacterial functionalities improved with increasing ethanol concentration before dipping drastically at 100% ethanol [6]. This could be attributed to the bactericidal mechanism of ethanol. While little is known about the specific bactericidal mechanism of ethanol, it is generally hypothesized that the solvent targets the cell membrane, causing catastrophic damage to the phospholipid layers, as well as intracellular proteins, leading to irreversible denaturation of these biomolecules [7]. As such, the inclusion of water molecules at 30% - 40% v/v helps to optimize the hydrophobicity-hydrophilicity balance of the ethanol, allowing better penetration of the chemical entity into and/or through the bacterial cell membrane [6].

Beyond the alcohol content, some alcohol-based sanitizers are also incorporated with an additional antimicrobial active ingredient, including chemical additives (e.g. chlorhexidine, cetylpyridinium chloride, etc.) as well as natural extracts (e.g. thyme leaf extract, chitosan, etc.) [7]. The addition of these secondary antimicrobials has been claimed to provide additional functionalities to the product including speeding up the rate of antimicrobial action and prolonging the period of antimicrobial protection. While many of the commercial sanitizers have made such claims regarding the additional benefits of a secondary active ingredient, there are no published literature that reports the impact of a secondary antimicrobial agent on the functionalities of sanitizers. This report provides a comprehensive study examining the bactericidal efficacy and functionalities of various alcohol-based sanitizers. A comparative analysis and discussion will also be conducted to evaluate the effect of adding a secondary antimicrobial agent on the overall functionalities of sanitizers.

## Materials and methods

All alcohol-based sanitizers used for the experiments were procured from a local pharmacy (Guardian, Singapore, Singapore) and an online platform (Shopee Pte Ltd, Singapore, Singapore). Chemicals and reagents were purchased from Sigma-Aldrich (Singapore, Singapore), unless otherwise stated.

Table 1 lists the alcohol-based sanitizers and the active ingredients incorporated within the respective products. Model bacterial strains used in this study include *Escherichia coli* UTI 89 (*E*. *coli*, and *Staphylococcus aureus* USA 300 (*S*. *aureus*).

**Table 1:** List of alcohol-based sanitizers and active ingredients incorporated within the different products. PBS was used as a negative control in the current study.

Sanitizer/Control	Active ingredients
Sanitizer A	70% Ethanol
Sanitizer B	62% Ethanol
Sanitizer C	70% Ethanol, Cetylpyridinium Chloride
Sanitizer D	70% Ethanol, Chlorhexidine
Sanitizer E	70% Ethanol, ULTRABactech^TM^
PBS (control)	-

### Zone of inhibition assay

Antimicrobial functionality of the respective sanitizers against both *E*. *coli* and *S*. *aureus* were evaluated qualitatively using a zone of inhibition assay [8]. Briefly, the overnight microbial suspension was sub-inoculated into fresh Mueller-Hinton (MH) broth and allowed to grow for 3 h to reach the exponential phase. The microbial suspension was harvested and diluted with fresh MH broth to a final concentration of 10^8^ CFU/mL. 1 mL of the bacteria suspension was then dropped and spread uniformly across the surface of an MH agar plate to form a uniform bacteria lawn. The plate was allowed to dry under atmospheric conditions (25°C) for 10 min. 10 uL of respective hand sanitizers was dropped onto the designated position of the plate. The culture plate was incubated overnight (18 h) at 37°C. The zone of inhibition was subsequently measured and recorded.

### Minimum Bactericidal Concentration (MBC) assay

MBCs of respective sanitizers against *E*. *coli* and *S*. *aureus* were determined using an established broth microdilution procedure [9]. Wells of a 96 well microplate were filled with 50.0 μL respective sanitizers at different dilution fators (1 x, 2 x, 4 x, 8 x, 16 x, 32 x, 64 x, 128 x) using serial dilution method. 50.0 μL of 10^6^ CFU/mL bacterial suspensions in MH broth were then added to the wells to obtain a final bacterial concentration of 5 x 10^5^ CFU/mL. The microplate was incubated at 37°C for 16 h. 5.0 μL of the resulting suspension from each well was plated on MH agar plate. The plates were subsequently incubated at 37°C for 18 h before determining the MBC through visual observation. MBC was determined to be the dilution factor at which no colony could be observed on the agar plate.

### Kinetics of bactericidal action

500 μL of respective sanitizers were added to an equal volume of 10^6^ CFU/mL of *S*. *aureus* suspension in MH broth. The mixtures were mixed thoroughly and incubated at 37°C. At respective time points (0 s, 15 s, 30 s, 1 min, 2 min, 3 min, 5 min, 10 min, 15 min, 30 min, 1 h, 2 h, 3 h, and 5 h), samples of the mixtures were withdrawn for CFU enumeration.

### Biofilm inhibition assessment

The capability of respective sanitizers in retarding biofilm formation, under a high microbial loading environment, was assessed based on an established assay [10]. Briefly, *S*. *aureus* was inoculated into rich Luria-Bertani (LB) broth and incubated overnight (18 h) at 37°C. Overnight bacteria culture was further diluted to a final concentration of 10^7^ CFU/mL. To each well of the 96-well microplate, 10 μL of respective sanitizers were added with 90 μL of bacteria suspension. The plate was subsequently incubated at 37°C for 18 h. The spent media was eventually removed. The used wells were then washed thrice gently with sterile phosphate-buffered saline (PBS) before gently tapping on a paper towel to dry the washed wells. 205 μL of 0.1% (w/v) crystal violet (CV) was added to each well and allowed to stain under gentle shaking at room temperature (25°C) for 30 min. The CV solution was eventually discarded and stained wells were washed gently with a copious volume of fresh PBS. The stained wells were then destained with 70% (v/v) ethanol for 30 min under atmospheric conditions (25°C) with gentle shaking. The absorbance of the solution was subsequently recorded at 595 nm.

### Sanitization effect of sanitizers on different types of surfaces

The instant sanitization capability of sanitizers on different types of material was evaluated using an assay, modified based on the JIS Z 2801 protocol [11]. Briefly, the surface of PDMS (surface area of 1 cm^2^) was inoculated with 50.0 μL of 10^6^ CFU/mL *S*. *aureus* culture. The inoculated PDMS were left to dry under atmospheric conditions (25°C) for 1 h. 30.0 μL of respective sanitizers were applied to the bacteria inoculated PDMS surface by the means of a nozzle spray. The treated materials were further left to incubate under atmospheric conditions (25°C) for a further 3 h. CFU enumerations were eventually conducted to assess the sanitization effect of the respective sanitizers. The assay was repeated for various candidature materials including plastic, aluminium, cotton-based fabric, and wood.

### Evaluating antimicrobial sustainability of sanitizers

A revised antimicrobial assay was conducted to evaluate the sustainability of antimicrobial protection that sanitizers can impart to applied surfaces. Briefly, ~30.0 μL of respective sanitizers were applied to 1 cm by 1 cm surface of PDMS using a spray nozzle and left to dry under atmospheric conditions (25°C) for 24 h. The treated surface was subsequently exposed to microbes by adding inoculating with 50 μL of 10^6^ CFU/mL *S*. *aureus* culture. The PDMS was incubated under atmospheric conditions (25°C) for 1 h before CFU enumeration of the material was conducted. The assay was repeated for various candidature materials including plastic, aluminium, cotton-based fabric, and wood.

### Statistical analysis

All experiments were conducted in triplicate (unless otherwise stated), with average and standard deviation calculated for all measurements. The non-parametric Mann-Whitney U-test was applied for analyzing differences in parameters. Statistical data analysis was conducted using Graphpad Prism (Version 6, Graphpad Software, Inc.).

## Results and discussion

### Antibacterial potency of sanitizers

Zone of inhibition [8,12] and minimum bactericidal concentration [9] are two gold-standard assays commonly used to assess the antibacterial potency of antimicrobial reagents. Table 2 highlights the results from the respective antibacterial assays for the tested alcohol-based sanitizers.

**Table 2:** Antibacterial potency assessment of various alcohol-based sanitizers using minimum bactericidal concentration and zone of inhibition.

Sanitizer	Dilution to minimum bactericidal concentration (x dilution)		Zone of inhibition (mm)	
	*E*. *coli*	*S*. *aureus*	*E*. *coli*	*S*. *aureus*
PBS	-	-	-	-
Sanitizer A	8.00 ± 0.00	8.00 ±0.00	8.67±0.58	-
Sanitizer B	8.00±0.00	8.00±0.00	-	-
Sanitizer C	8.00±0.00	≥256.00±0.00	13.33±0.58	20.00±0.00
Sanitizer D	≥256.00±0.00	≥256.00±0.00	21.67±0.58	22.33±0.58
Sanitizer E	8.00±0.00	64.00±0.00	12.00±0.00	13.67±0.58

Zone of inhibition represents a qualitative method to investigate the antimicrobial potency of an antimicrobial compound. The presence of a distinct clear zone of inhibition is usually indicative of potent antimicrobial functionality. Comparing the different alcohol-based sanitizers, sanitizer A and B do not possess a clear zone of inhibition, while the rest of the sanitizers showed a distinct clear zone. This could be attributed to the lack of a secondary antimicrobial agent in their formulations. While ethanol has been known to possess good sterilization functionality, due to its volatility, it vaporizes rapidly, resulting in it being unable to reach bacteria that have penetrated further into the nutrient agar matrix. In contrast, with an additional antimicrobial agent, ethanol and the secondary active ingredient can work synergistically to target and kill bacteria deeper within the gel matrix, resulting in a distinct zone of inhibition for Sanitizer C, D, and E. The difference in the diameter of the clearance zone could largely be attributed to the differences in antibacterial potency of the respective secondary antimicrobial agents. Chlorhexidine possesses the best antibacterial potency followed by cetylpyridinium chloride and naturally derived ULTRABactech^TM^. A comparative study by *Suller et al*. showed corresponding results with chlorhexidine illustrating a better antibacterial potency than cetylpyridinium chloride against various *S*. *aureus* and *Enterococcus* [13]. On the other hand, natural antimicrobials generally possess a weaker antibacterial potency in comparison to their chemical counterparts, accounting for the smallest zone of inhibition for Sanitizer E, with a natural antimicrobial agent (ULTRABactech^TM^) [14]. Besides antimicrobial potency, other factors, such as density of bacteria lawn and physicochemical properties of the antimicrobial compound can also have direct impacts on the zone of inhibition. A study by *Abbas et al*. demonstrated the effect of electrostatic charge on the resulting zone of inhibition. An increase in silver nanoparticle charge can significantly increase the clearance zone [15]. A separate study by *Agitha et al*. highlighted that particle size can also impact the zone of inhibition. An increase in particle size resulted in a lower zone of inhibition due to the higher resistance of the particles to move through the tightly weaved agar matrix [16].

MBC assay represents a more standardized method to assess the antimicrobial potency of antimicrobial compounds, with microbial concentration, media, and volume-controlled [9]. The quantitative result from the MBC assay corresponds to that of the qualitative zone of inhibition assay. Sanitizer A and B, which consist of only ethanol, demonstrated the lowest antibacterial potency. The addition of a secondary antimicrobial agent generally enhances the bactericidal functionality of sanitizers, allowing a higher dilution factor before reaching MBC. Among sanitizers with secondary antimicrobial agents, Sanitizer D, which contains chlorhexidine, demonstrated the highest antibacterial potency with MBCs of ≥256x dilutions against both *E*. *coli* and *S*. *aureus*. Sanitizer C and E differed in their antibacterial potency against *S*. *aureus*, with Sanitizer C (MBC ≥256x dilution) being more effective than Sanitizer E (MBC = 64x dilution). As aforementioned, the lower MBC of Sanitizer E could be attributed to the weaker antimicrobial functionality of natural antimicrobial that was used as the secondary antimicrobial agent [14].

Despite differences in terms of antibacterial potency, it is evident that alcohol-based sanitizers with secondary active ingredients generally possess a better bactericidal functionalities as compared to sanitizers that contain only 70% alcohol.

### Bactericidal kinetics of alcohol-based sanitizers

More than antibacterial potency, it is crucial that sanitizers are capable of eliciting their bactericidal action rapidly, ideally within minutes of application [4]. A bactericidal kinetics comparative study was conducted to evaluate the speed of antibacterial action between the respective alcohol-based sanitizers.

**Fig 1:**
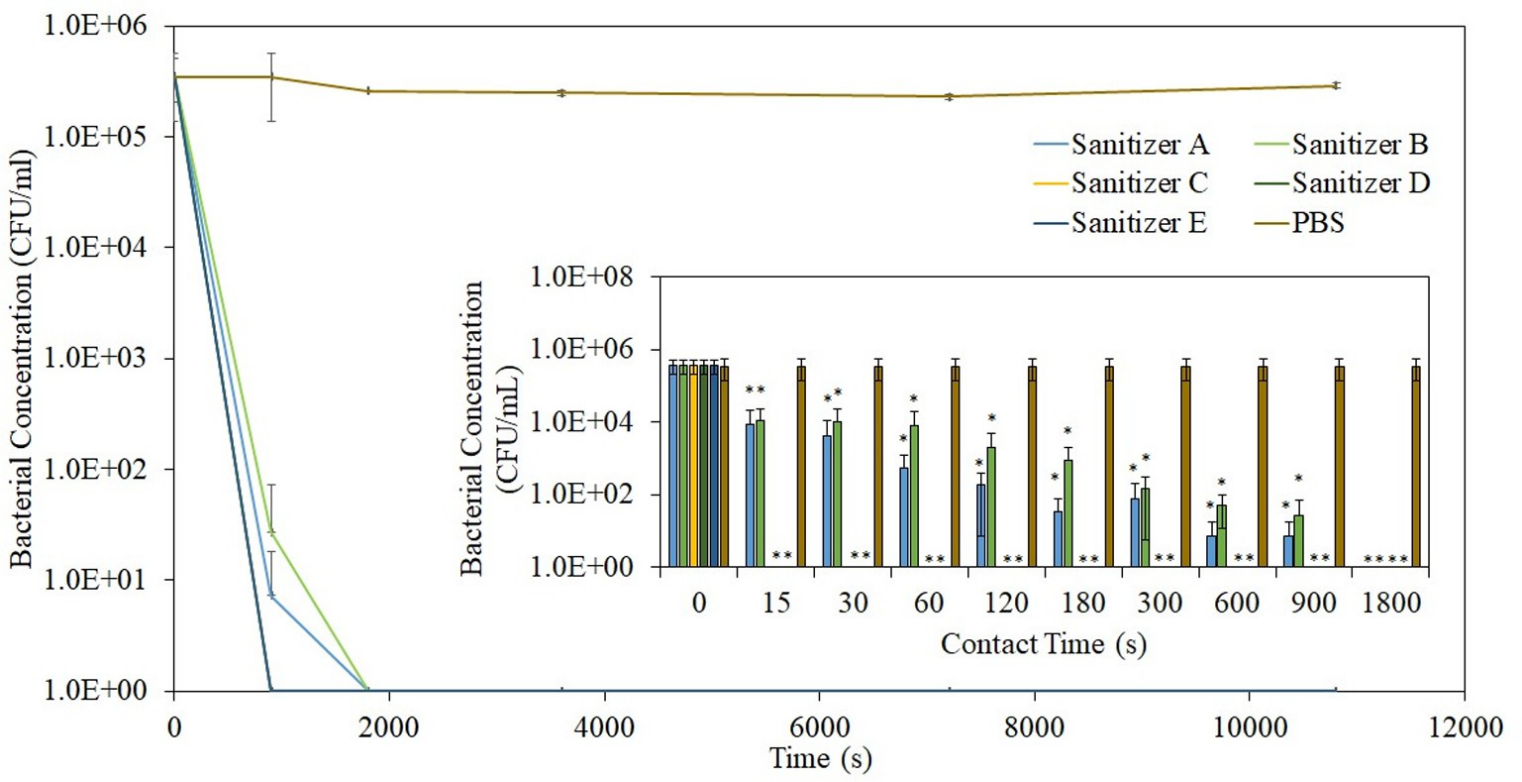
Kinetics of bactericidal activities for different alcohol-based sanitizers.

All sanitizers demonstrated rapid antibacterial action, eliminating the microbes within 30 min of exposure (Fig 1). In comparison to sanitizers containing only 70% alcohol, alcohol-based sanitizers with secondary antimicrobial agents (i.e. Sanitizer C, D, and E) possessed faster bactericidal kinetics, effectively targeting and killing 5x10^5^ CFU/mL of *S*. *aureus* within 15 s of contact. Sanitizers containing only 70% alcohol illustrated a slower killing efficacy, requiring up to 1800 s to completely kill all the inoculated bacteria. The secondary antimicrobial agent worked synergistically with the primary agent (i.e. 70% alcohol) to enhance the bactericidal efficacy. While not specifically for sanitizers, the enhancement effect of such combinatorial therapy has been well reported for antibiotic treatment. An *in vivo* study by *Sande et al*. showed that *S*. *aureus* in infected rabbit models was eradicated in half the time when treated with the gentamicin-penicillin combination as compared to penicillin or gentamicin monotherapy [17,18].

A closer study of the bactericidal kinetics for Sanitizer A and B showed that Sanitizer A has a slightly faster bactericidal activity. This could be attributed to the higher concentration of ethanol in Sanitizer A. An established study by *Harry et al*. showed that increasing ethanol concentration from 25% to 30% led to an enhancement in bactericidal kinetics against *Pseudomonas aeruginosa* from 3 min to 40 s respectively [19].

### Anti-biofilm functionality of sanitizers

**Fig 2:**
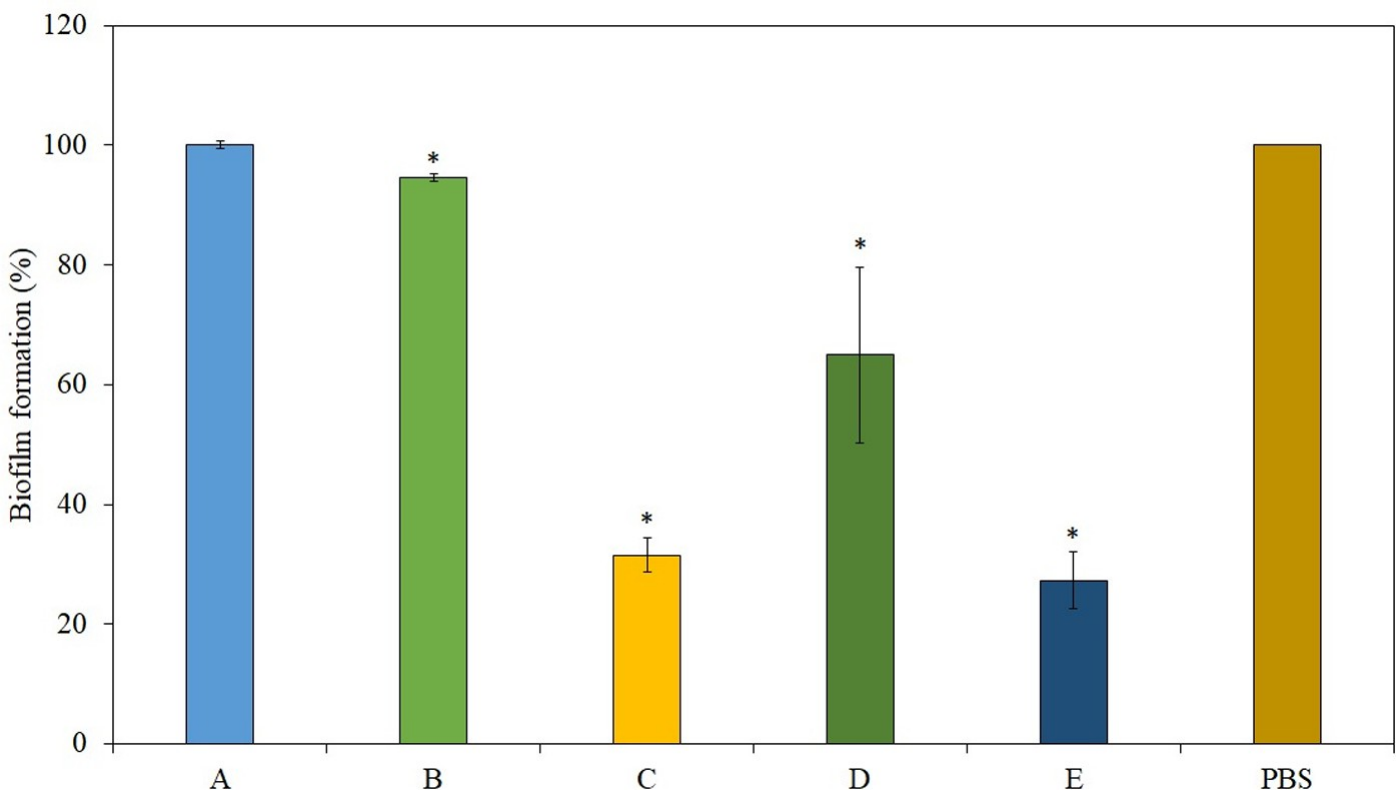
Anti-biofilm functionality of different alcohol-based sanitizers. * indicates P < 0.05 as compared to the control (PBS).

Biofilm establishment of a surface is often a consequence of a material being inhabited by microbes for a prolonged period [20]. While frequent application of sanitizer on surfaces can greatly reduce the probability of such occurrence, there is still a chance for microbial attachment and biofilm formation on the surface. This is especially the case under high microbial loading conditions (10^7^ CFU/mL and beyond), which goes beyond the antimicrobial capability of the sanitizers. A biofilm inhibition assay was conducted to assess the capability of the respective alcohol-based sanitizers in deterring biofilm formation under high bacterial loading. Fig 2 illustrates the extent of biofilm formation on model polystyrene surface with different alcohol-based sanitizers under high microbial loading of 10^7^ CFU/mL. Surfaces treated with Sanitizer A and B showed a similar degree of biofilm establishment as that treated with PBS control. On the other hand, Sanitizers C, D, and E showed a significantly lower degree of biofilm formation. The result highlights the benefits of including a secondary antimicrobial agent in the sanitizer formulations. The volatile nature of ethanol meant that most of the alcohol will vaporize within minutes, causing the alcohol content in the suspension to drop below its effective concentration. This is especially disadvantageous in such prolonged assays, where the leftover microbes are allowed time to attach and proliferate on the surface. Unlike alcohol, the secondary antimicrobial agent is normally a non-volatile compound, being able to remain on the surface and/or suspension after the alcohol has evaporated, actively deterring opportunistic microbial attachment and growth. The presence of an additional antimicrobial agent also allows the sanitizer to target and kill a larger population of inoculated microbes. These factors combine to aid in resisting microbial attachment and biofilm formation on surfaces treated with alcohol-based sanitizers with secondary antimicrobial agents.

### Applicability of sanitizer for different surfaces

**Fig 3:**
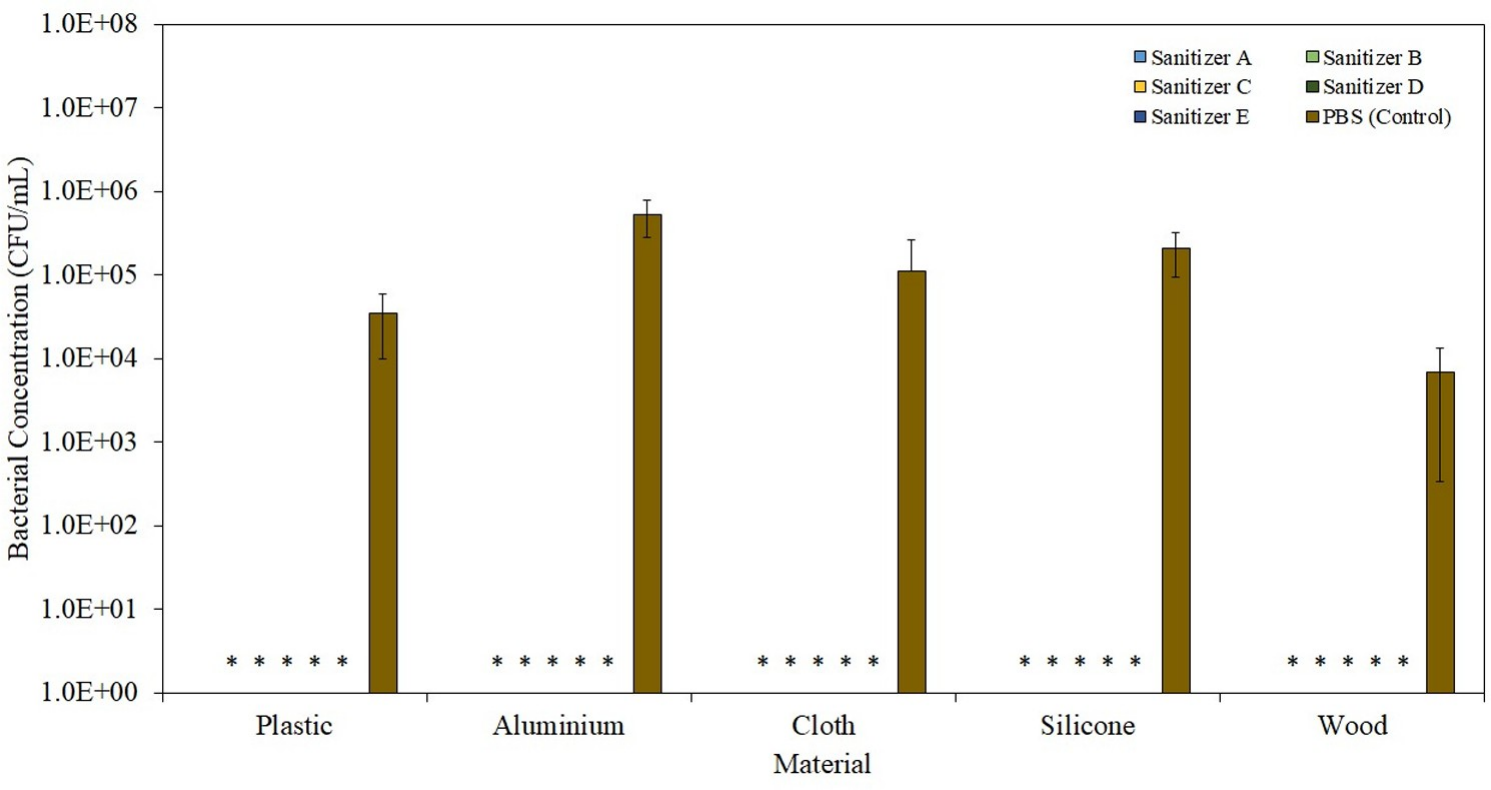
Instant sanitization functionality of respective alcohol-based sanitizers on different types of materials and surfaces. * indicates P < 0.05 as compared to the control (PBS).

Beyond the application of hands, alcohol-based sanitizers are also being constantly applied to different materials, to clean the respective surfaces. Fig 3 highlights the instant sanitization functionality of respective alcohol-based sanitizers on different material surfaces. All tested alcohol-based sanitizers, regardless of whether they contained secondary antimicrobial agents or the type of surfaces, were able to elicit the instant sanitization effect, killing up to 99.99% of all the surface inoculated *S*. *aureus*. Such potent antibacterial results of alcohol-based sanitizers on different surfaces are of no surprise and have been validated in various publications [21,22]. In a recent study by *Hi-Ryun Lee et al*., the authors showed that 70% ethanol is capable of eliciting a 5 log_10_ reduction on all types of fabric including silk, Tencel, cotton, etc [22]. Similarly, when *Chambers et al*. treated bacteria-infested plastic with 70% ethanol, all microorganisms were killed after 1 h [21]. Due to the bactericidal mechanism of action of 70% ethanol, the type of surface will not have any significant impact on its antibacterial effect.

### Antibacterial sustainability of alcohol-based sanitizers

Beyond instant sanitization, there is a demand for sanitizers to not only provide instant sanitization but also impart prolonged and sustained antimicrobial protection (i.e. to offer continuous antimicrobial protection for a certain period after application) on the treated surface. A simple testing protocol, based on JIS Z 2801 standard, was developed to assess the antimicrobial sustainability of the respective alcohol-based sanitizer. Briefly, sanitizer-treated surfaces were left under atmospheric conditions for 24 h before being exposed to 10^6^ CFU/mL of bacteria. After 3 hours of contact time, the bacteria suspension was harvested for CFU enumeration to evaluate the extent of antibacterial sustainability of the respective treated surfaces.

**Fig 4:**
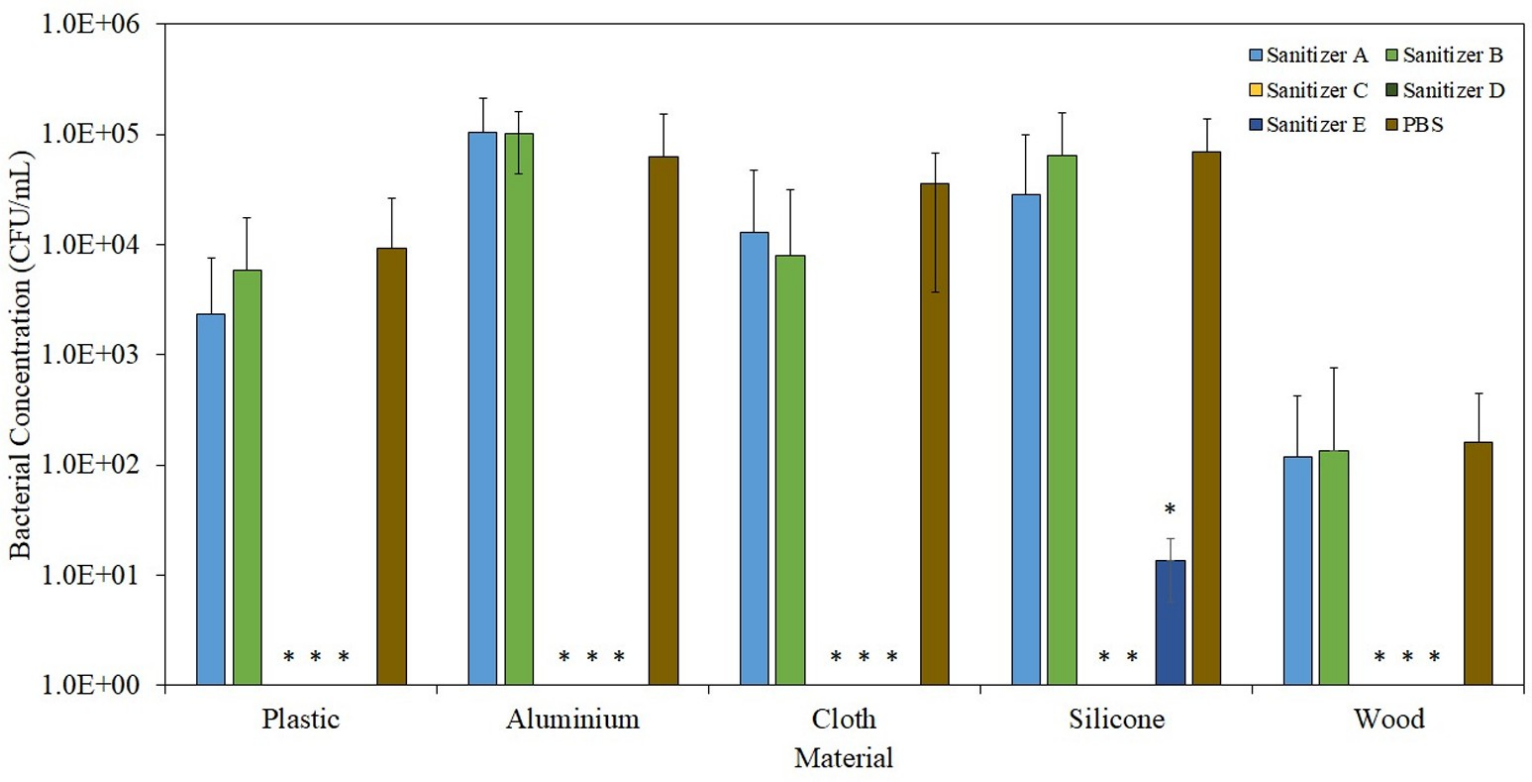
Sustained antibacterial protection (24 h) offered by different surfaces treated with different sanitizers. * indicates P < 0.05 as compared to the control (PBS).

As illustrated in Fig 4, surfaces treated with Sanitizer C, D, and E demonstrated sustained antibacterial protection, being able to target and kill ≥99.9% of the opportunistic bacteria that lands on the surface 24 h after treatment. On the other hand, Sanitizers A and B did not impart such sustained antibacterial protection to the surface. Such differences can be largely attributed to the inclusion of a secondary active ingredient in Sanitizer C, D, and E. For alcohol-based sanitizers that do not contain a secondary active ingredient (e.g. Sanitizer A and B), once the ethanol vaporizes, which usually occurs within minutes after application, the treated surface is again exposed to the threat of opportunistic microbial contamination. In the case of alcohol-based sanitizers with the secondary active ingredient, as the ethanol vaporizes, the secondary active ingredient remains behind as residue, forming a thin protective layer on the treated surface. Opportunistic microbes that subsequently land on the surface will be actively targeted and eradicated by the remnant secondary active ingredient. Corresponding results were reported in a study by *Sidney et al*., where the authors compared the antibacterial sustainability of DAB hand sanitizer (containing 0.12% benzalkonium chloride as an active ingredient) to a comparator hand sanitizer (containing 63% ethyl alcohol) [23]. Volunteers treated with DAB hand sanitizer demonstrated persistent antibacterial protection, eliciting a 3–4 log_10_ reduction of the bacteria 4 h after initial application. In contrast, volunteers treated with comparator product did not reduce bacterial viability by even 1 log_10_. The authors hypothesized that such antibacterial sustainability is probably due to the benzalkonium chloride remaining on the surface of the skin after drying. Similar results were observed in a separate study by *López-Gigosos et al*., where a significant reduction factor (≥ 2.0) was recorded for triclosan-alcohol and chlorohexidine-alcohol sanitizers after 90 min, while a negligible reduction factor (≤ 1.0) was recorded for propanol, mecetronium-alcohol and soap sanitizers [24]. Similarly, such sustained antibacterial activity can be attributed to the residual secondary active ingredient that remains on the treated surface.

While the addition of a secondary active ingredient may seem to improve the antibacterial sustainability of sanitizers, the extent and longevity of such effect are still very much dependent on various factors including the stability of the antimicrobial agent and external environmental conditions. In the same study by *López-Gigosos et al*., the authors highlighted that while antimicrobial sustainability was significant on wet inert surfaces treated with triclosan-alcohol and chlorohexidine-alcohol sanitizers, the effect was much less pronounced on dry surfaces [24]. The result demonstrated that external conditions such as dryness of the surface can adversely impact the antimicrobial performance of the residual active ingredient. It is also worthy to note that while Sanitizer E has demonstrated good antibacterial persistency, most natural antimicrobials are quite vulnerable to processing conditions, solvents, and external environments. This is more of a unique case where the natural antimicrobial agent is highly stable. In a recent study by *Thamnopoulos et al*., the antimicrobial stability of milk samples formulated with different concentrations of bee propolis was evaluated. While effective in the first instance, the antimicrobial functionality of propolis dwindled with increasing days of storage. On top of that, the rate of degradation is much faster when the respective milk formulations are stored at 10°C as compared to that stored at 4°C [25]. The drop in antimicrobial functionality could be due to the propolis degrading gradually due to exposure to less optimal environments. This represents an exemplary study to highlight the sensitivity of natural bioactive to external environmental conditions.

## Conclusion

All alcohol-based sanitizers have been demonstrated to be effective in providing basic instant sanitization functionality. Similarly, all tested alcohol-based sanitizers possess rapid antibacterial action, effectively targeting and killing 5x10^5^ CFU/mL of microbes within 30 min. The addition of a secondary active ingredient proved to be beneficial to enhance the effectiveness and functionality of alcohol-based sanitizers by (1) improving the kinetics of antibacterial action, (2) imparting good anti-biofilm properties to the treated surface preventing opportunistic microbial attachment, proliferation, and biofilm establishment, and (3) providing sustained antibacterial protection to the treated surface. While the addition of a secondary active ingredient seemed to be beneficial, it is important to note that the physicochemical properties of the secondary active ingredient as well as the external environment can affect the extent of its performance. As such it is important to evaluate the stability of the active ingredient before deciding which antimicrobial agent to incorporate into their formulation.
